# Myocardial perfusion imaging SPECT left ventricle segmentation with graphs

**DOI:** 10.1186/s40658-025-00728-5

**Published:** 2025-03-10

**Authors:** Ádám István Szűcs, Béla Kári, Oszkár Pártos

**Affiliations:** 1 Computer Algebra, Eötvös Loránd University, Pázmány Péter blvd. 1/c, Budapest, Pest, 1117 Hungary; 2 Nuclear Medicine, Semmelweis University, Üllői street 78b, Budapest, Pest, 1083 Hungary

**Keywords:** Single-photon emission computed tomography (SPECT), Myocardial perfusion imaging (MPI), Image segmentation, Optimization, Flow graphs

## Abstract

****Purpose**:**

Various specialized and general collimators are used for myocardial perfusion imaging (MPI) with single-photon emission computed tomography (SPECT) to assess different types of coronary artery disease (CAD). Alongside the wide variability in imaging characteristics, the apriori “learnt” information of left ventricular (LV) shape can affect the final diagnosis of the imaging protocol. This study evaluates the effect of prior information incorporation into the segmentation process, compared to deep learning (DL) approaches, as well as the differences of 4 collimation techniques on 5 different datasets.

****Methods**:**

This study was implemented on 80 patients database. 40 patients were coming from mixed black-box collimators, 10 each, from multi-pinhole (MPH), low energy high resolution (LEHR), CardioC and CardioD collimators. The testing was evaluated on a new continuous graph-based approach, which automatically segments the left ventricular volume with prior information on the cardiac geometry. The technique is based on the continuous max-flow (CMF) min-cut algorithm, which performance was evaluated in precision, recall, IoU and Dice score metrics.

****Results**:**

In the testing it was shown that, the developed method showed a good improvement over deep learning reaching higher scores in most of the evaluation metrics. Further investigating the different collimation techniques, the evaluation of receiver operating characterstic (ROC) curves showed different stabilities on the various collimators. Running Wilcoxon signed-rank test on the outlines of the LVs showed differentiability between the collimation procedures. To further investigate these phenomena the model parameters of the LVs were reconstructed and evaluated by the uniform manifold approximation and projection (UMAP) method, which further proved that collimators can be differentiated based on the projected LV shapes alone.

****Conclusions**:**

The results show that prior information incorporation can enhance the performance of segmentation methods and collimation strategies have a high effect on the projected cardiac geometry.

## Introduction

SPECT data is inherently stochastic, which renders the interpretability and automatic detection techniques challenging. In myocardial perfusion protocols the ultimate goal is to find plausible CAD. With the high specificity and non-invasive nature, different cardiac conditions can be successfully characterized [1]. Application of a specific radiotracer at stress and rest stages, quantitative measurement of the left ventricle is possible to be obtained.

The patient is carefully positioned in the camera after administering the necessary radiopharmaceutical and the SPECT data is acquired $12 \div 16$ minutes before getting further processed [2]. With the large choice of nuclides, one can functionally describe the target tissue.

Before segmentation, the projection data is inspected for proper technical aspects of the acquisition, patient motion artifacts, attenuation, scatter and background effects, as well as organ overlaps. As a next step the projection frames are back-projected quantitatively to get accurate becquerel (Bq) per each reconstructed volume milli liter (mL) element. Based on the clinical assessment protocol the acquisition, reconstruction, and segmentation can vary, therefore the current paper focuses on perfusion studies. In MPI one has to solve the reorientation problem, which involves the segmenting of, and finding the orientation, of the left ventricle based on the midline of the LV cavity. The paper discusses the former, known as segmentation or classification.

In the simplest cases automating the segmentation of LV in MPI SPECT volumes can be a challenging problem due to the low resolution and the soft edges caused by the nature of the imaging protocol [3]. To further complicate algorithmic solutions many additional real-life artifacts can mislead the automation [4]. Indirectly higher noise levels have effects on partial volume effect (PVE) because they demand stronger filtration methods for evaluation, resulting in higher PVE. Most can be hepatic, intestinal, or breast attenuation, whereas patient motion can lead to significant imaging artifacts as well. The sources are diverse and difficult to handle in automated scenarios, holding potential for further improvement over current solutions.

Various commercial solutions already exist to segment the SPECT MPI left ventricles, e.g. Corridor 4DM [5], Cedars-Sinai [6], Emory Cardiac Toolbox [7] and the Yale approach [8]. Most of these approaches utilize either deep learning, the level-set method (LSM), K-means clustering, or deformable model (DM)s. These methods have their applicability in different cases [9], where it was found that most of the methods still require different level of supervision, which is often caused by the large variability in imaging conditions and protocols. Most of these approaches haven’t been tested yet on a multi-vendor and a newer multi-camera setup to assess their robustness against different patient variability and in varying conditions.

**Fig. 1:**
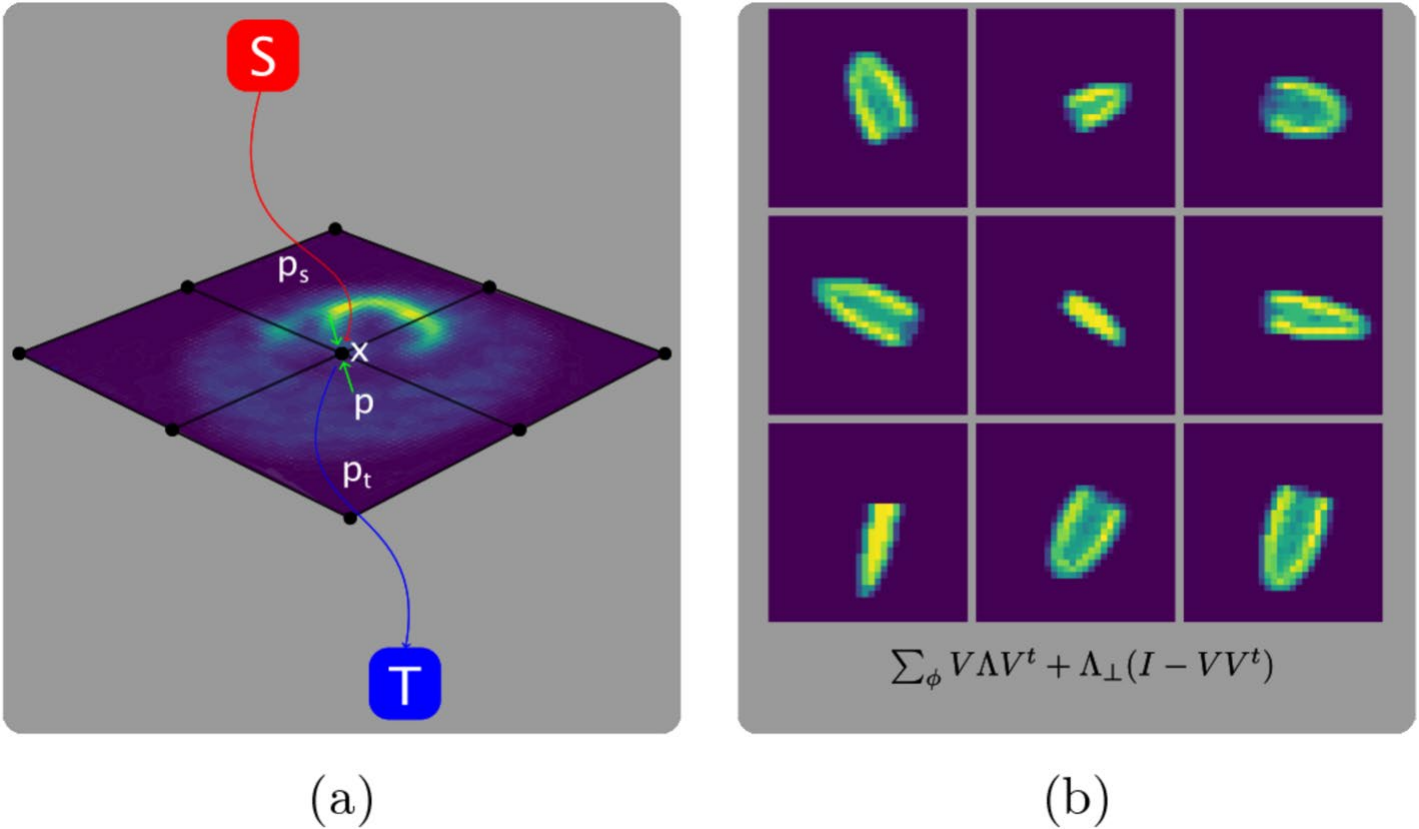
Schematic overview about different stages of the approach. The subfigures depict the main contributions. **a** Illustrates the continuous min-cut algorithm, applied on a reconstructed slice of an MPI LV image. **b** Shows the statistical-based approach to handle prior information about the functional mapping of the left ventricle

In this paper, a novel shape prior with a geometric optimization method, named advanced segmentation method (ASM) is introduced to solve the classification problem in perfusion studies, as schematically demonstrated in Fig. 1. The prior information is formulated in an energy minimization, spatially continuous graph setting [10–12]. The method originates from the well-known Mumford-Shah functional [13], which is defined as the following

1 $$\begin{aligned} E[J,B]&= \alpha \int _{D}(I({\vec {x}})-J({\vec {x}}))^{2}\,\textrm{d} {\vec {x}}\\ &\quad +\beta \int _{D/B}{\vec {\nabla }}J({\vec {x}})\cdot {\vec {\nabla }}J({\vec {x}})\,\textrm{d} {\vec {x}}\\ &\quad +\gamma \int _{B}ds,  \end{aligned}$$

where *I*, *J* is the image and its’ model, *D* is the domain of definition, *B* is the set of the boundaries, $\alpha , \beta , \gamma$ are hyperparameters and $\nabla$ is the nabla vector differential operator. Knowing that this functional is non-convex, therefore it is impossibly hard to compute. To make it numerically attainable, the following approach [14] is taken to create a relaxed model

2 $$\begin{aligned}&\min _{\lambda (x) \in [0, 1]} \int _{\Omega } (1 - \lambda (x))C_{s}(x) dx + \int _{\Omega } \lambda (x)C_{t}(x) dx\\ & \quad \quad + \alpha \int _{\Omega } |\nabla \lambda (x)| dx,  \end{aligned}$$

where $\lambda (x) \in [0, 1]$ is proven to result in a convex relaxed approximation. This relaxation comes with further statements, such as the following lemma

### Lemma 1

(Truncation lemma) If $\lambda \in K$ solves Eq. (2), then so does $\lambda ^{t}$ for almost all $t \in [0, 1]$.

Here $K = \{ \lambda \in BV(\Omega ), 0 \le \lambda (x) \le 1, \forall x \in \Omega \}$ BV is a set of functions with bounded variation (BV) and

3 $$\begin{aligned} \lambda ^{t}(x) = {\left\{ \begin{array}{ll} 1\ \text {if}\ \lambda (x) > t\\ 0\ \text {otherwise} \end{array}\right. } x \in \Omega . \end{aligned}$$

## Materials and methods

### Notation and problem formulation

The mathematical abbreviations are shown in Table 1. For three-dimensional setting, let $\Omega$ be a closed 3-D domain, and *s*, *t* be the source and sink terminal vertices of a graph. At every $x \in \Omega$, let *p*(*x*) be a passing, $p_{s}(x)$ a directed source from *s* to *x* and $p_{t}(x)$ a directed sink flow from *x* to *t* as it can be seen in Fig. 1a. With this setup, the primal constraints are the following

4 $$\begin{aligned} |p(x)|&\le C(x),\ \forall x \in \Omega \end{aligned}$$

5 $$\begin{aligned} p_{s}(x)&\le C_{s}(x),\ \forall x \in \Omega \end{aligned}$$

6 $$\begin{aligned} p_{t}(x)&\le C_{t}(x),\ \forall x \in \Omega \end{aligned}$$

7 $$\begin{aligned} \nabla \cdot p(x)&= p_{s}(x) - p_{t}(x),\ \text {a. e.}\ x \in \Omega . \end{aligned}$$

Here *C*(*x*) stands for the capacity of a virtual pipe or edge at *x* spatial position in the graph, where further subscripts stand for source and sink flow capacity constraints. $\nabla \cdot$ is the divergence operator and a.e. stands almost everywhere, which is a measure-theoretic term for a weak sense of inclusion, except on a possible subset of a zero measure. Further transforming the problem one arrives at the *primal-dual model*

8 $$\begin{aligned}&\sup _{p_{s}, p_{t}, p} \inf _{\lambda } \left\{ \int _{\Omega } (1 - \lambda )p_{s} + \lambda p_{t} + \lambda \nabla \cdot p\ dx \right\} \nonumber \\ &\quad \quad \text {s.t.}\ p_{s}(x) \le C_{s}(x),\ p_{t}(x) \le C_{t}(x), |p(x)| \le C(x), \end{aligned}$$

where let the objective function of Eq. (8) be $E(p_{s}, p_{t}, p; \lambda )$. Knowing the former *primal-dual* formulation suffices all the conditions of the minimax theorem, the min and max operators can be interchanged. With this, it can be shown that, optimizing the *primal-dual* model is equivalent to the *primal* model, known as the continuous max-flow

9 $$\begin{aligned}&\min _{\lambda (x) \in [0, 1]} \int _{\Omega } \left\{ (1 - \lambda (x))C_{s}(x) \right. + \lambda (x)C_{t}(x) \\ &\quad \quad \left. + C(x)|\nabla \lambda (x) | \right\} dx,  \end{aligned}$$

which can be formulated based on Eq. (8) as the following

10 $$\begin{aligned} L_{c}(p_{s}, p_{t}, p, \lambda )&= \int _{\Omega } p_{s} dx \\ &\quad + \int _{\Omega } \lambda (\nabla \cdot p - p_{s} + p_{t}) dx \\ &\quad - \frac{c}{2}\Vert \nabla \cdot p - p_{s} - p_{t}\Vert , \end{aligned}$$

where $c > 0$ and it is optimized with the multiplier-based algorithm, described further by Yuan [10] in alg. 5.1. One of the core steps of the algorithm is the total variation (TV) minimization of

11 $$\begin{aligned} p^{k+1} = \Pi _{\alpha }\left( p^{k} + c \nabla ( \nabla \cdot p^{k} - F^{k} ) \right) , \end{aligned}$$

where $\Pi _{\alpha }$ is the convex projection. The method is based on Chambolle’s projection algorithm [15], which projects Eq. (11) onto the convex set $C_{\alpha } =\{q\ |\ \Vert q\Vert \le \alpha \}$.

**Table 1:** Mathematical abbreviations

non-convex	Optimization problems having cost functions with non-convex characteristics
BV	Set of bounded variation (BV) , consists of real-valued functions having finite total variation (TV)
TV	Total variation is the higher dimensional analogue of the absolute value in one dimension
primal-dual	An optimization problem possessing dual and primal characteristics too. The aim is to lower the dual-gap between the primal and dual formulations of the problem
minimax	A rule to minimize the loss in the worst case possible in an optimization problem
Mahalanobis	Distance measures that are computing the length between a point and a probability distribution
KL	Kullback-Leibler (KL) divergence, measures how much a model probability distribution differs from a true probability distribution. Asymptotically KL minimization amounts to maximum likelihood estimation
CV	Chan-and-Vese (CV) dataterms stand for methods in active contour field which minimize the difference between the mean inner and outer activities

Given that a globally optimal solution can be attained with the CMF algorithm, one would think that it is possible to segment the left ventricle from the volumetric 3-dimensional data. However, as mentioned before, the SNR levels are quite low and multiple artifacts hinder the performance of any classification method. To overcome the challenges, prior information can be implemented in the optimization process to prefer particular objects of specific shape(s) [16].

### Shape priors

For shape prior information to be incorporated in the target energy functional, various techniques can be used to overcome the problem of occlusion or large noise in general. Most approaches use explicit shape priors, which demand a clear and precise description of the prior information of the object. In [19] and [20] a rather new approach is introduced, which is based on a staged approach to incorporate the shape prior information in the optimization process. The method is based on a Riemannian geometry-based gradient descent on the Lie group of the similarity transformations of the geometric prior. The resulting problem is no longer convex and the explicit optimization of the transformation parameters can result in trapped local optima.

To overcome the aforementioned problems, one can approach the solution with nonlinear statistical shape priors as described in [21]. The method works as follows: given a set, *m* number of training data in ${\mathbb {R}}^{n}$, the technique tries to estimate the “mean” shape in a higher-dimensional feature space *Y*. Given that the corresponding energy is the negative logarithm of the probability, a Mahalanobis-type distance can be formulated in the feature space *Y* as follows

12 $$\begin{aligned} E_{\phi }(z) = {\tilde{\phi }}(z) \Sigma ^{-1}_{\phi } {\tilde{\phi }}(z). \end{aligned}$$

Since the number of training data shapes is lower than the dimensionality of the problem, the covariance matrix will be degenerate, where the zero eigenvalues can be replaced with a constant $\lambda _{\perp }$

13 $$\begin{aligned} \Sigma _{\phi } = V \Lambda V^{t} + \lambda _{\perp }(I-VV^{t}), \end{aligned}$$

where $\lambda _{1} \le \dots \le \lambda _{r}$ are the eigenvalues and $V_{1}, \dots , V_{r}$ are the corresponding eigenvectors. Knowing the form of $\Sigma _{\phi }$, the eigenvectors lie in the span of the training data

14 $$\begin{aligned} V_{k} = \sum _{i=1}^{m} \alpha _{i}^{k} {\tilde{\phi }}(z_{i}),\ 1 \le k \le r. \end{aligned}$$

Combining Eqs. (13) and (14) one arrives at the form of the following energy

15 $$\begin{aligned} E_{\phi }(z)&= \sum _{k=1}^{r} \left( \sum _{i=1}^{m} \alpha _{i}^{m} {\tilde{k}}(z_{i}, z) \right) ^{2} \cdot (\lambda _{k}^{-1} - \lambda _{\perp }^{-1})\\ &\quad +\lambda _{\perp }^{-1} \cdot {\tilde{k}}(z, z),  \end{aligned}$$

where $z_{i}$ is the i-th training sample, ${\tilde{k}}$ is a kernel of choice. With this approach Eq. (15) can result in a method invariant to affine transformations by converting the argument *z* to its mean.

### Left ventricular model

**Fig. 2:**
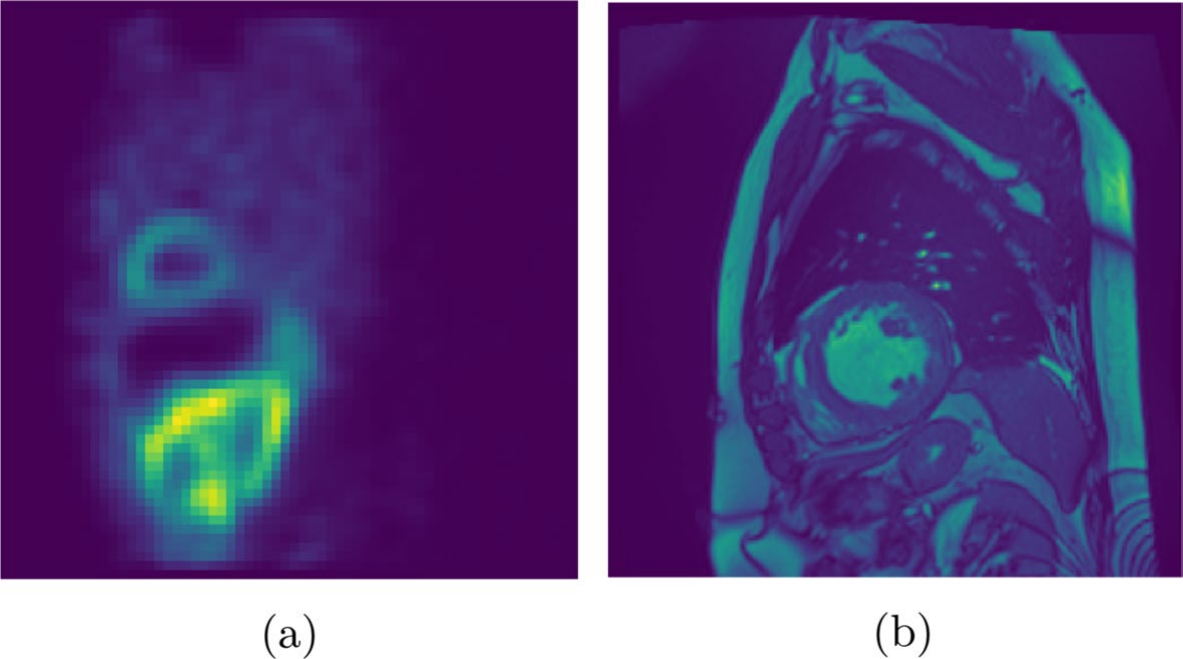
Difference between functional (SPECT) (**a**) and anatomical (MR) (**b**) [17, 18] left ventricles in SA orientation

Building shape priors for MPI SPECT data is a challenging task. The problem comes from the inherent characteristics of the imaging protocol. During rest or stress perfusion imaging, the cardiac, thoracic, and “whole-body” motion can further distort the final reconstructed volumes. Combining these facts with low-resolution and high-sensitivity cameras, the resulting image hardly resembles an anatomic modality’s cardiac data, see Fig. 2.

**Fig. 3:**
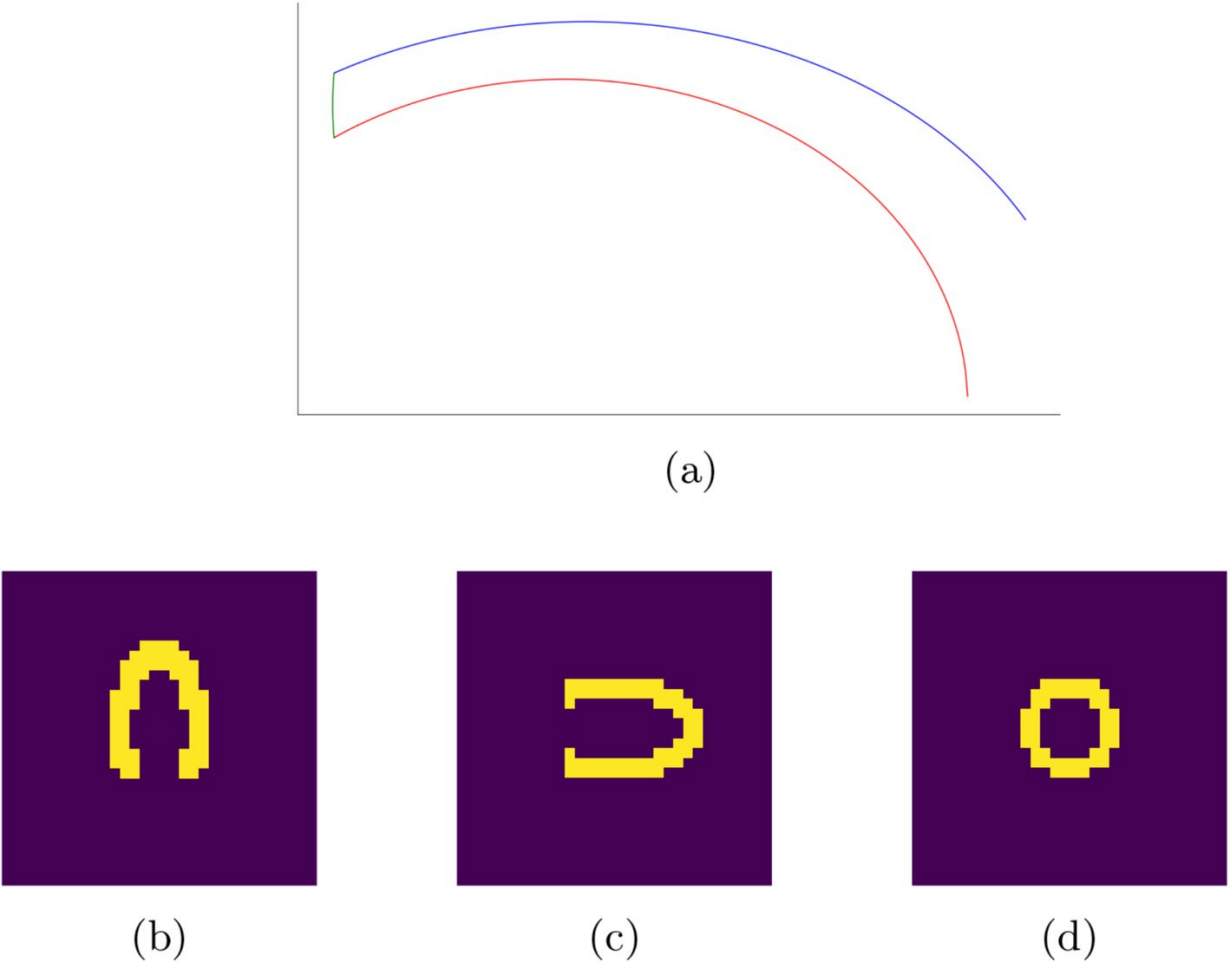
Cardiac shape prior for SPECT myocardial perfusion imaging. **a** Shows the profile curves of the shape prior, where the different colors encode the various controllable curves of the endo, epicardial, and septal surfaces. The final prior is derived from the profile curves by rotating them in 3D. The lower row images show the shape prior in all the clinical orientations, horizontal long axis (HLA) (**b**), ventricular long axis (VLA) (**c**), and short axis (SA) (**d**)

To address the big difference between the functional (SPECT) and anatomical (CT, MR) left ventricles, a new approach is taken to handle the geometrical prior information incorporation into the classification process. As seen in Fig. 3 the model is built by having the least amount of parameters to handle the large variety of left ventricular appearances. The shape information is the following

16 $$\begin{aligned} c_{{\rm epi}}(u)&= a \cdot \sqrt{1 - (u^{2} / c^{2})} \end{aligned}$$

17 $$\begin{aligned} c_{{\rm endo}}(v)&= b \cdot \sqrt{1 - (v^{2} / d^{2})} \end{aligned}$$

18 $$\begin{aligned} c_{{\rm sep}}(w)&= (\kappa \cdot (w - \text {epi}) \cdot (w - \text {endo}))^{-1}, \end{aligned}$$

where $c_{{\rm epi}}$ is the epicardial surface curve, $u \in [\sigma , c]$. The endocardial surface curve is $c_{{\rm endo}}$, $v \in [\sigma , d]$ and $c_{{\rm sep}}$ is the septal curve, between the epi and endocardial endpoints. To get the 3D model of the aforementioned curves, the entire object is rotated around the x-axis.

### Shape and data interaction term

To handle the interaction between the data and shape terms, the optimization follows the steps of [19], where, in order to keep the truncation property of the cost functional, the structure is the following

19 $$\begin{aligned}&\inf \left\{ J(u) + \frac{\lambda + \mu }{2} [ \langle 1 - u, (I_{{\rm eff}} - c_{0})^{2} \rangle \right. \\ &\quad \quad \left. + \langle u, (I_{{\rm eff}} - c_{1})^{2} \rangle ] \right\} ,  \end{aligned}$$

where $I_{{\rm eff}} = \frac{\lambda }{\lambda + \mu } I + \frac{\mu }{\lambda + \mu } I_{prior}$ is the convex combination of the effective and observed image domain and $u \in \Omega \rightarrow \{0, 1\}$ is a binary label function. In this setting Eq. (19) can be solved with the Chan-and-Vese (CV)-algorithm in a continuous cuts setting.

As for the data-term, by the nature of the SPECT data, ${\mathbb {L}}_{2}$ norm-based approximations can not handle the Poisson noise well, as described in [22]. Therefore a more statistically fit model is needed. One approach is to utilize the Kullback–Leibler (KL) divergence in combination with CV data terms [23], where

20 $$\begin{aligned} KL_{p_{i} \Vert p_{o}}(u) = \int _{\Omega } p_{i}(x | u) \log {\left( \frac{p_{i}(x | u)}{p_{o}(x | u) }\right) }. \end{aligned}$$

Here $p_{i}, p_{o}$ are the inside and outside probability density functions. This model is denoted as (KLCV), which, based on the computed tests, expressed a lot better performance compared to the simple CV functional optimization.

### Quantitative evaluation

The segmentation accuracy was evaluated on the following metrics. Accuracy = $\frac{TP + TN}{TP + TN + FP + FN}$, Precision = $\frac{TP}{TP + FP}$, Recall = $\frac{TP}{TP + FN}$, Intersection over Union (IoU) = $\frac{TP}{TP + FN + FP}$, DICE coefficient = $\frac{2 TP}{2TP + FP + FN}$, where T stands for true, F false, N is negative, P is positive.

### Implementation

The code^1^ is written in PyTorch [24] and models were trained both on a single Nvidia A100 GPU [25] and a 1080Ti GPU. The annotated data consists of 80 patients with reconstructed complete field-of-view (FOV) volumes. The implementation uses the TorchIO [26], Scikit-learn [27], Point Cloud Utils [28] package, and the GeomLoss [29] library extensively for the Sinkhorn loss and differentiation of cost functions.

### Data

For the noise tolerance test, simulated data was used to carry out the results, which consisted of mathematical phantoms generated with x-cat [30].

Patient data consisted of 5 different groups, totaling 80 patients. **(A)** A black-box dataset with mixed geometries, pharmaceuticals, and acquisitions. This group included 40 patients, of which 27 were female, and 13 male. The average patient age was 69.62 years, with an average height 167.25 cm, and weight 78.11 kg. **(B)** A different group was created on the latest multi-pinhole (MPH) geometry with APT73 collimators on the Mediso AnyScan Trio camera. This group consisted of 10 patients, 7 females and 3 males with an average age of 68.88 years, height of 169.66 cm, and weight of 73.8 kg. **(C)** A comparative LEHR-HS collimator was placed on the Trio camera summing up 10 patients total, 8 females and 2 males with an average age of 68.0 years, height of 176 cm, and weight of 92.0 kg. **(D)** To compare the latest cameras and their geometries with those used currently, older machines were also included in the study. Mediso CardioD acquisitions consisted of 10 patients: 1 female, and 9 males with an average age of 75.6 years, height of 151 cm, and weight of 71.0 kg. **(E)** Patients were screened through the Mediso CardioC camera, 7 females and 3 males. The retrospective data were in “interfile” format, which only enables to determine the average age of 70.33 years.

### Acquisition

Patient data acquisition was performed on different versions of Mediso AnyScan, AnyScan Trio SPECT and SPECT/CT systems. CT Image scanning parameters: SPIRAL acquisition type; CT Dose Index Volume 0.22; tube voltage 150 kVp; and tube current 30–50 mAs. myocardial perfusion SPECT (MPS) studies were performed at 4 participating sites with various isotopes: $^{99m}$Tc MIBI, $^{99m}$Tc Tetrofosmin and $^{201}$TI Chloride. Acquisitions were performed with both 64 and 128 matrices, with 64 projections on a two-headed camera and 96 projections on a triple-headed camera. The angular interval was 180 on two-headed, CardioC, CardioD cameras, while 360 for the triple-headed cameras. The studies with attenuation maps were reconstructed with the TeraTomo method [31]. Samples without Attenuation Correction (AC) utilized the ordered subset expectation maximization (OSEM) reconstruction method [32] to create the complete FOV volume stack.

### Labeling

Delineating the gold standard left ventricular volume from complete FOV images is a challenging task. The labeling was conducted by nuclear cardiology professionals on all the 80 patients. To segment and check myocardial limits, 3D Slicer [33] was used, by utilizing the local region growing method followed by manual trimming and addition of correct LV voxels.

## Results

### Quantitative evaluation on phantom data

This part of the test methodology entailed the evaluation of the ASM method on the phantom dataset. To evaluate the noise tolerance of the methodology, simulated phantom data was generated by the x-cat [30] program . Projection frames were created from the volumetric x-cat data and then back-projected—reconstructed—with the Tera Tomo method. The target model was a male patient, with a small beating to generate the best approximation of real perfusion data.

**Fig. 4:**
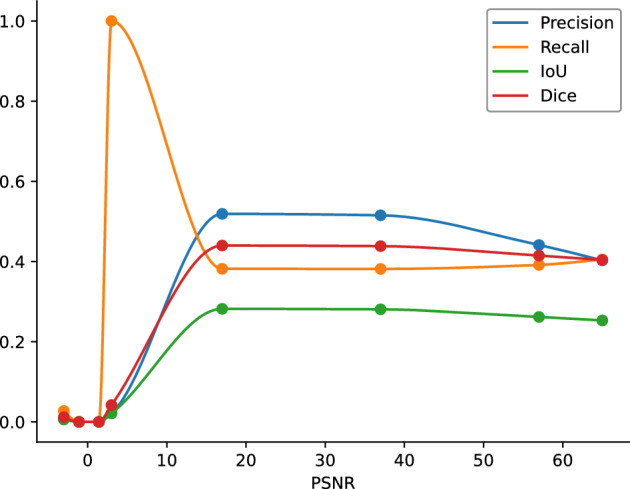
Demonstrating the robustness against different noise levels

To mimic a real SPECT background, different levels of random Poisson noise were added to the simulated left ventricular image, as can be seen in Fig. 4. As it is required at lower levels of peak signal-to-noise-ratio (PSNR), the method behaves poorly, around 0.0 in all the quantitative metrics. Around and above $\approx$5 it becomes a lot better and stays flat as intended. One anomaly is the recall measure, which hits the highest levels after the threshold of 5, which could be interpreted by knowing that the data term in the segmentation is based on the KL divergence, finding the ideal balance between the interior and exterior of the segmenting region.

### Qualitative evaluation on patient data

**Fig. 5:**
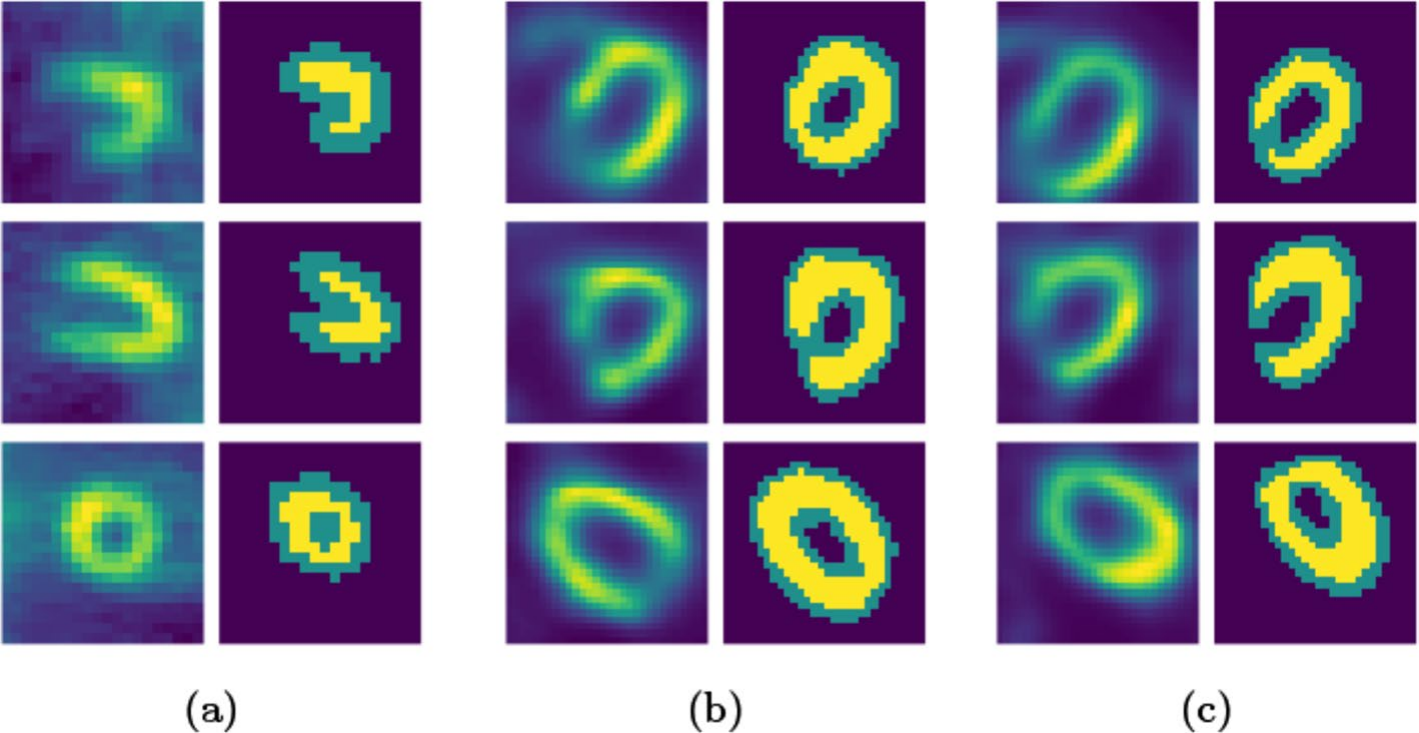
Different experiments on various geometries. The first column is patient data in the transaxial, sagittal, and coronal orientations. The outline—epicardial area—of the segmented left ventricles is shown in green and the inside—myocardial, endocardial—are in yellow. The second column consists of the predicted LV voxels in their respective orientation. **a** Depicts segmentation results on parallel collimation geometry. The first column of **b** shows results on an 8-min acquisition with MPH collimation, after stress. On **c** performance on the same collimation and acquisition as the latter, but after rest period in the protocol

The next step in the testing methodology consisted of patient data from two geometries, one is the most common one: the parallel collimation, and the other is the bleeding edge MPH collimator. ASM showed promising results both on the parallel and the MPH datasets. The interpretation of Fig. 5 is that the shape prior information can help the segmentation process to classify the pixels more accurately than simply applying a data term. Since the prior information is built from the statistical ensemble of different left ventricles characterized by the cardiac model, and the parameters were identically chosen for the different geometry segmentations, one might find more optimal parameters to segment the different geometries, which is investigated later in the paper.

### Quantitative evaluation on patient data

To be able to get a better understanding of the performance of the method and to investigate the characteristics of different collimation and acquisition protocols, the evaluation of patient data consisted of two different approaches. One was to estimate the mean power of the algorithm on a black-box dataset, denoted as (A) in the mechanism. This survey aimed to get a good estimation of how the ASM method compares to different approaches [34, 35] in segmenting different left ventricles with mixed collimation, pharmaceuticals, and reconstruction methods. As depicted in Table 2, the ASM algorithm can outperform both competing techniques, only in recall does it fall behind a little bit, which could be described by the model parameters in creating the segmentation statistics in kernel space. Since the parameters for the preferred left ventricles are created by uniformly selecting parameters from the model control manifold, one might be able to find a better choice of parameters or pose a higher weight on the shape parameters in the optimization process.

**Table 2:** Averaged segmentation results on the mixed, patient, and simulated dataset. Our approach is capable of outperforming [34] and [35] in most of the metrics

Averaged metric performance on mixed geometries				
	Precision	Recall	IoU	Dice score
ASM (A)	**0.6920**	0.7086	**0.5358**	**0.6561**
Adam et al. (A)	0.4123	**0.8934**	0.1273	0.1005
Zhu et al. (A)	0.0285	0.8131	0.0291	0.0682

Bold values represent the best results in each of the metrics

**Table 3:** Segmentation results on the different geometries. The mean and the standard deviation are computed on each group of data

Averaged metric performance on different acquisition geometries				
	Precision	Recall	IoU	Dice score
MPH Stat. (B)	0.4294 ± 0.2512	0.6974 ± 0.3187	0.3341 ± 0.1728	0.4607 ± 0.2367
CardioD (D)	0.5234 ± 0.4044	0.5051 ± 0.3956	0.2653 ± 0.2278	0.3663 ± 0.3292
CardioC (E)	0.4025 ± 0.0908	0.6701 ± 0.2164	0.3311 ± 0.0860	0.4790 ± 0.1294
Parallel Coll. (C)	0.4323 ± 0.2478	0.5929 ± 0.2979	0.3000 ± 0.1625	0.4246 ± 0.2417

After evaluating the methods on the dataset (A), the ASM was used to investigate further questions on the differentiability of left ventricles between collimation geometries and their traits. To answer and investigate this, the algorithm was evaluated on all the (B)–(C)–(D)–(E) datasets by computing the mean and standard deviation of each quantitative metric. As demonstrated in Table 3, the results show a mixed picture of left ventricles under different collimation. The results having been interpreted, it seems that their optimum can be reached on the MPH collimation, which, however, shows large deviations in both recall as well as precision. On the older geometries, CardioC and CardioD, similar results can be seen. And lastly, with the parallel geometry, the result looks mostly similar that of the former two collimation strategies. This raises a further question: whether the acquired and back-projected left ventricles “behave” differently or similarly in the interpretation of the ASM method.

**Fig. 6:**
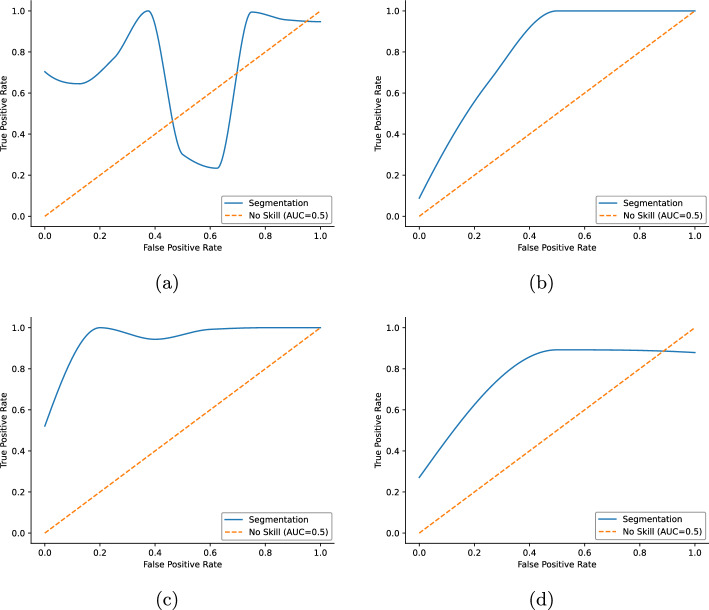
Plots expressing the power of the segmentation method as a binary classifier on different projection techniques. ROC curves of the different collimation geometries. **a** Depicts the MPH, **b** is the CardioC, **c** is the CardioD and **d** is the parallel collimator. It can be understood as the cummulative distribution function of the segmentation probability on different collimator geometries

The interpretation of the different receiver operating characterstic (ROC) curves of Fig. 6, is the following. As it is seen in Fig. 6a, the MPH APT73 collimator fluctuates in performance between a good classifier and a random one. Having further investigated this data, it was found that the acquisition strategies had been different inside this group. One half was screened with a step-and-shoot helical trajectory, whereas a stationer collimator positioning was used for the other half. The performance of the stationer acquisition was far superior to the helical acquisition, which introduced the fluctuation in the ROC curve. A better stability—a smoother curve—is shown in Fig. 6d. However, after a certain point, its performance deteriorates. The best curves are produced with CardioD and CardioC collimation techniques, shown in Figs. 6b and c. The cause of this great stability could be two-fold: both geometries are optimized for cardiac imaging, with the reconstruction method as well, which produces well-delineated and articulated left ventricular volumes with a minimal background noise.

### Perfusion defect impact on segmentation performance

Datasets (A)–(B)–(C)–(D)–(E) had various levels of perfusion defects across all patients. The mixed-geometry black-box dataset (A) had $27.87\%$ mean defect in the left ventricles with $7.72 \%$ standard deviation. MPH dataset (B) had $28.45 \%$ mean defect in the myocardial volume with $5.07 \%$ standard deviation. The parallel geometry dataset (C) had $38.94 \%$ mean and $3.04 \%$ standard deviation function deficit. For the CardioD (D) dataset, it consisted of left ventricles with $31.87 \%$ mean defect with $2.27 \%$ standard deviation. The last, CardioC dataset (E) had $35.77 \%$ mean and $4.12 \%$ standard deviation defects across the patients.

**Fig. 7:**
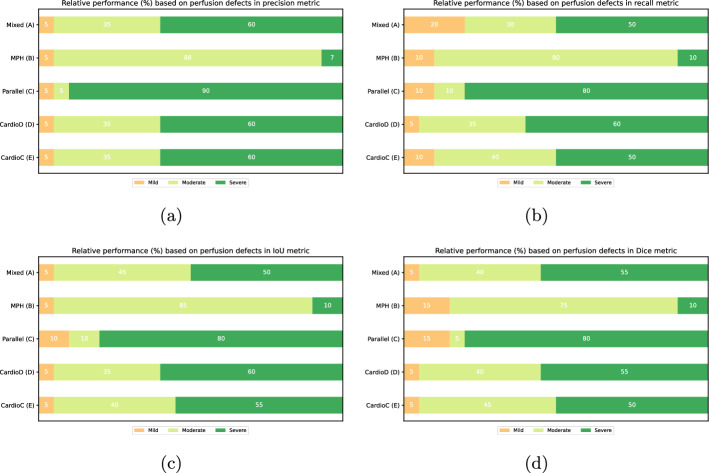
Relative segmentation performance on the datasets with different perfusion defects. **a** Shows the performance in precision, **b** recall, **c** IoU and **d** dice metrics

During evaluation the patients were distributed into three groups. Mild defects with less than $20 \%$ defect size, Moderate with defect sizes between $20 \%$ and $35 \%$ and Severe with larger than $35 \%$. The segmentation method was evaluated based on the grouping on all the different geometries and datasets. As shown on Fig. 7 perfusion defects had various impact on the segmentation performance with the shape prior. Analyzing the results, it is shown, that patients with mild or no perfusion defects are underrepresented compared to moderate and severe cases. On (A)–(D)–(E) mixed-geometry, CardioD and CardioC datasets the performance between mild and sever cases are balanced. Whereas on parallel (C) geometries, the method outperformed the moderate cases in severe ones. As for on the MPH (B) the algorithm reached much better results on moderate cases than in any other group. The distribution of various perfusion cases of left ventricles can be described by the fact that, patients often administered at the nuclear cardiology clinics with proven or possible previous cardiac events, rendering low perfusion defects scarce in the datasets.

### Finding differences between left ventricles under various collimation geometries

As it has been demonstrated, left ventricles “behave” differently under various collimation geometries. To further explore this effect, a deeper analysis was conducted on the myocardial volumes of the (B)–(C)–(D)–(E) datasets.

First, so as to characterize the differences, statistical tests were executed on the segmented datasets of each geometry. The setting was the following: having segmented every myocardium in each group, the outline—i.e. surface—of the left ventricles was computed with the marching cubes algorithm. Then the unbalanced Sinkhorn distances were calculated with a $\varepsilon = 1e-3$ blur. After each distance matrix had been computed per geometry, a Wilcoxon signed-rank test was performed in a one-sample test on the upper triangular part of the matrix.

**Fig. 8:**
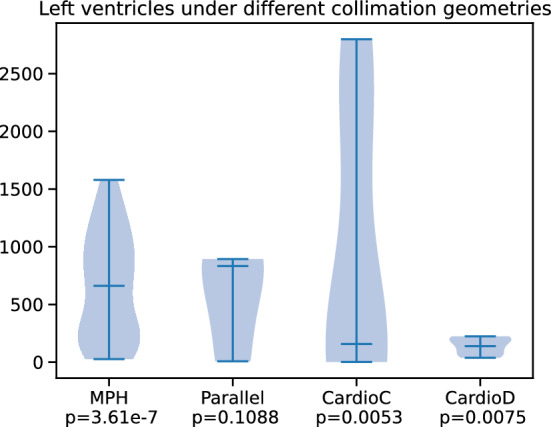
Wilcoxon signed-rank test between the surfaces of each segmented left ventricle per geometry. The *p* values depict the significance of each test

Evaluating the left ventricles in this setting further proves the previous finding, namely that the differences between collimation geometries can be characterized. As seen in Fig. 8, the results are expansive. On MPH geometries the left ventricles look the least “alike”, therefore hearts loaded with different pharmaceuticals will look distinguishable. The second-best significance was shown on both CardioC and CardioD geometries, both projecting the data well. One interesting finding was in the case of Parallel geometries, which showed a worse significance in differentiating left ventricles. This lies in the field of reconstruction. CardioD and CardioC used specific optimized reconstruction methodologies, whereas Parallel utilized a different, less controllable procedure, which caused most of the hearts to “thicken”, making every left ventricle look the same-ish as per the statistical tests with entropic regularized Wasserstein distances.

The findings on the segmented left ventricles were interesting, but there was one more approach worth mentioning. Knowing that the left ventricles can be differentiated on each of the geometries, it is interesting to estimate what the manifold of parameters in the cardiac models looks like. And if it is possible to describe it, what conclusion can be drawn from it in a clinical setting? In order to conduct such an investigation, one needs to reconstruct the left ventricle parameters from the feature space, which is equipped with an Euclidean geometry. The derivation of the algorithm can be found in the Appendix. To be able to estimate the manifold of parameters a new approach, UMAP [36] was used.

**Fig. 9:**
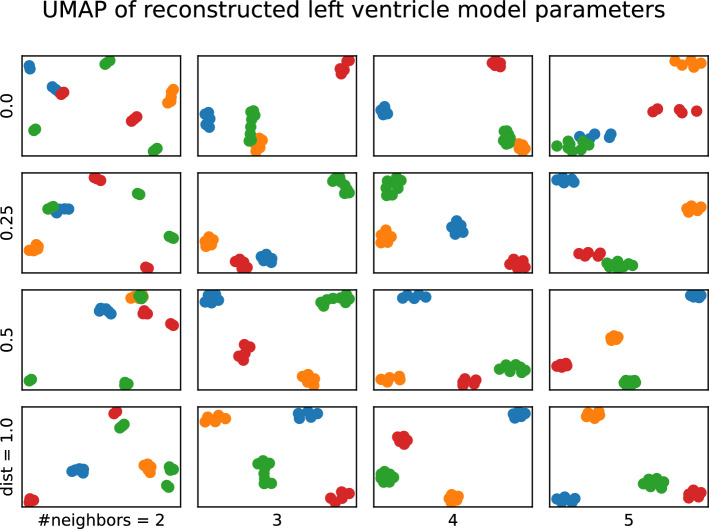
UMAP estimation on the manifold of the cardiac model parameters. Blue depicts the MPH, orange the parallel, green the CardioC, and red the CardioD geometry parameters

After the reconstruction of the cardiac model parameters, the result of the UMAP estimation can be seen in Fig. 9. As per previous evaluations, the cardiac models on different collimation geometries can be distinguished according to their parameters. Lowering the distance and the neighbor counts the MPH as well as the CardioD and CardioC geometries look similar, as opposed to when increasing these parameters of the UMAP estimation, the collimation geometries produce separable left ventricular model parameters.

## Discussion

In this paper a new approach is introduced: the segmentation of the left ventricle from myocardial perfusion SPECT. The novelty comes from the combination of a continuous max-flow approach with a density estimation based shape prior information, which can get quite challenging in the case of SPECT complete FOV data. A statistically motivated low-dimensional model is introduced, which has proven to help the classification task in diverse left ventricular volumes.

Further findings:

1. As it was seen in [34], challenging cases can drive the self-supervised learning (SSL) approach away from an ideal performance. To overcome this, the current study proves that involving prior information about the left ventricular shape in a statistical shape prior setting can enhance the classification performance. These findings were supported by both qualitative and quantitative measurements as seen in Fig. 5 and Table 2.
2. By knowing that, the myocardial segmentation can be enhanced with statistical shape priors, a further, a more constituent analysis was conducted to get a better understanding of the behavior of different collimation geometries in perfusion imaging. As a first test, 4 groups of different reconstructed acquisitions were created and evaluated quantitatively. As seen in Table 3, the best performance can be reached on MPH APT73 collimators. However , one must approach the problem carefully by choosing the better acquisition trajectory, stationer, and a well-optimized reconstruction. Aside from the performance of the bleeding edge collimator, it was conjectured that specialized cameras, such as CardioC and CardioD, can become superior, see in Fig. 6 when it comes to stability and left ventricle delineation. The causality of this comes from the physical setting of the camera and the well-controllable reconstruction parameters especially focused on cardiac imaging.
3. To get an even better understanding of the segmented left ventricles, statistical tests further proved the conjecture of the differentiability of cardiac images under various collimations. The MPH geometry showed great statistical significance in separating different left ventricles, as well as the specialized CardioC, CardioD cameras. However, as seen on Parallel collimators, this can deviate if the reconstruction method is unoptimized for myocardial imaging.
4. Further, evaluating the method, the perfusion defect impact analysis showed that on (A)–(C)–(D)–(E) datasets, the algorithm reached the highest performance on severely defected left ventricles. On MPH (B) the best results has been achieved on moderate cases.
5. The last finding showed that, based on the reconstructed left ventricular parameters in the interpretation of the model statistics, resulting in a distinguishable manifold by the collimation geometries. As seen in Fig. 9, this shows that every geometry has a large influence on how one must interpret the image at hand when it comes to myocardial perfusion imaging.

## Conclusions

The contribution presented here is mainly focused on increasing the quantitation performance of the myocardial perfusion imaging protocol. Major findings were: (1) A prior information based algorithm, can model a wide variability of left ventricular shapes. Whereas the method can outperform DL approaches, it gives a more interpretable structure about the underlying shapes. (2) With the applicability of this methodology it was feasible to distinguish different projection geometries solely from the segmented left ventricles. This further helps in the understanding of the perfusion defects under different collimation, therefore derived inter-collimation based errors can be avoided. (3) Consequently, this approach raises the importance of normal database creation for each geometry and collimation technology.

## Data Availability

The datasets generated and/or analysed during the current study are not publicly available due data protection act but are available from the corresponding author on reasonable request and with permission of Semmelweis University.

## Abbreviations

SPECT: Single-photon emission computed tomography
MPI: Myocardial perfusion imaging
TPD: Total perfusion deficit
EF: Ejection fraction
LEHR: Low energy high resolution
FOV: Field-of-view
CAD: Coronary artery disease
MPH: Multi-pinhole
CT: Computed tomography
TV: Total variation
SNR: Signal-to-noise-ratio
KL: Kullback–Leibler
LV: Left ventricle
LVR: Left ventricular
MLEM: Maximum-likelihood expectation maximization
OSEM: Ordered subset expectation maximization
OT: Optimal transport
DL: Deep learning
ROC: Receiver operating characterstic
UMAP: Uniform manifold approximation and projection
LSM: Level-set method
DM: Deformable model
ASM: Advanced segmentation method
CMF: Continuous max-flow
CV: Chan-and-Vese
VLA: Ventricular long axis
HLA: Horizontal long axis
SA: Short axis
MR: Magnetic resonance
PSNR: Peak signal-to-noise-ratio
SSL: Self-supervised learning
KLCV: Kullback–Leibler Chan-and-Vese
AC: Attenuation Correction
MPS: Myocardial perfusion SPECT
ML: Machine learning
GAN: Generative adversarial network
RL: Representation learning
VIT: Vision transformer
BV: Bounded variation
PVE: Partial volume effect

## Footnotes

1 https://github.com/JacksonFurrier/ejnmmi_2024_code.git.
